# The poor accuracy of D-dimer for the diagnosis of prosthetic joint infection but its potential usefulness in early postoperative infections following revision arthroplasty for aseptic loosening

**DOI:** 10.1186/s12879-022-07060-8

**Published:** 2022-01-27

**Authors:** M. Fernandez-Sampedro, I. Sanlés-González, C. García-Ibarbia, N. Fañanás-Rodríquez, M. Fakkas-Fernández, M. C. Fariñas

**Affiliations:** 1 Infectious Diseases Department, Hospital Universitario Marqués de Valdecilla, University of Cantabria, IDIVAL, Av. Valdecilla s/n 39008, Santander, Spain; 2 Internal Medicine Department, Hospital Universitario Marqués de Valdecilla, University of Cantabria, Santander, Spain; 3 Clinical Analysis Department, Hospital Universitario Marqués de Valdecilla, Santander, Spain; 4 Orthopaedic Surgery Department, Hospital Universitario Marqués de Valdecilla, Santander, Spain

**Keywords:** Plasma D-dimer, Prosthetic joint infection, PJI guidelines, First-line screening test

## Abstract

**Background:**

D-dimer was introduced in 2018 as an alternative biomarker for C-reactive protein (CRP) in the diagnostic of prosthetic joint infection (PJI) criteria of the Musculoskeletal Infection Society. We assessed the accuracy of plasma D-dimer for the diagnosis of early, delayed, and late PJI according to Infectious Diseases Society of America (IDSA) criteria, and whether persistently high levels of D-dimer in cases of aseptic loosening (AL) may be predictive of subsequent implant-related infection.

**Methods:**

A prospective study of a consecutive series of 187 revision arthroplasties was performed at a single institution. Septic (n = 39) and aseptic revisions (n = 141) were classified based on IDSA criteria. Preoperative assessment of CRP, erythrocyte sedimentation rate (ESR) and D-dimer was performed. Receiver operating curves were used to determine maximum sensitivity and specificity of the biomarkers. The natural progress of D-dimer for AL cases was followed up either until the date of implant-related infection at any time during the first year or 1 year after revision in patients without failure. Clinical outcomes for those AL cases included infection-related failure that required a new surgery or need for antibiotic suppression.

**Results:**

Preoperative D-dimer level was significantly higher in PJI cases than in AL cases (p = 0.000). The optimal threshold of D-dimer for the diagnosis of PJI was 1167 ng/mL. For overall diagnosis of PJI, C-reactive protein (CRP) achieved the highest sensitivity (84.6%), followed by erythrocyte sedimentation rate (ESR) and D-dimer (82% and 71.8%, respectively). Plasma D-dimer sensitivity was lower for all PJI types. When combinations of 2 tests were studied, the combined use of ESR and CRP achieved the best accuracy for all types of PJI (76.9%). 4.25% of AL cases had implant failure due to implant-related infection during the first year after the index revision arthroplasty, only the cases with early failure maintained high D-dimer levels.

**Conclusions:**

Plasma D-dimer did not offer an improvement over the individual or combined diagnosis for any type of PJI according to IDSA criteria. Persistently raised levels of D-dimer after revision arthroplasty in AL cases might be used to effectively diagnose early postoperative infection.

**Supplementary Information:**

The online version contains supplementary material available at 10.1186/s12879-022-07060-8.

## Introduction

In recent years, several scientific societies have developed criteria to standardize definitions for prosthetic joint infection (PJI) [1–4]. However, none have been widely adopted and the diagnostic approach to patients with suspected PJI remains extremely variable from center to center, depending on local experience, technological equipment, and adherence to available guidelines.

The main peripheral blood parameters used for the preoperative diagnosis of PJI include primarily 2 serological markers, serum C-reactive protein (CRP) and erythrocyte sedimentation rate (ESR). In the last 2 years, D-dimer has been recommended as a promising biomarker in suspected PJI, and has, in fact, been included in the 2018 Musculoskeletal Infection Society (MSIS) and International Consensus Meeting (ICM) criteria [5, 6]. Relevant studies that used MSIS or ICM criteria addressing the evaluation of D-dimer for the diagnosis of PJI reach different conclusions [7–12]. This may be due to the different methodologies used among the studies and, because the thresholds of serological markers may vary, depending on the heterogeneity of the definitions, the time of infection, different D-dimer assays, and/or the infecting organism, therefore more work is needed to further validate their role in the diagnosis of PJI [6]

No published data are available on the role of D-dimer, taking into account the Infectious Diseases Society of America (IDSA) guidelines which describe the combined use of an abnormal ESR and CRP as the best combination for identifying patients with suspected PJI. In this study, we assessed the accuracy of plasma D-dimer in improving the preoperative diagnosis of early (less than 3 months after prosthesis implantation), delayed (between 3 and 12 months), and late PJI (> 12 months after the prosthesis implantation) according to IDSA criteria, and whether the persistence of high levels of D-dimer in cases of aseptic loosening (AL) may be predictive of subsequent implant-associated infection.

## Materials and methods

In this prospective observational study, all consecutive patients aged 18 years or older undergoing a total or partial revision of knee or hip arthroplasty were enrolled between February 2013 and February 2015. The study was conducted in the Division of Orthopedics of the Hospital Universitario Marques of Valdecilla, a tertiary care hospital.

Patients were identified as having PJI according to IDSA guidelines [1]. Patients with a non-infectious diagnosis were defined as cases of AL: pain in the thigh or hip region, knee pain and radiological signs of loosening (inadequate initial fixation, mechanical loss of fixation over time, or biologic loss of fixation caused by particle-induced osteolysis around the implant). All patients with previous arthroplasty revisions were performed for non-infectious causes.

Since ESR, CRP and white blood cell count are part of our clinical routine; a preoperative determination was performed 2 weeks before the surgery. Based on receiver-operating-characteristic (ROC) analysis of the data, the cut-off level for CRP was > 1 mg/dl, while for ESR it was > 15 mm/h. D-dimer levels were evaluated in heparin plasma samples using the Tina-quant D-Dimer immunoturbidimetric assay (Roche Diagnostics©) on the Cobas c501 analyzer (Roche©) [13].

The histopathological criteria established by Morawietz and Krem (Additional file 1: Appendix S1) [14] were used to define a standardized evaluation of the periprosthetic membrane. Venous blood samples were obtained preoperatively on the day of surgery, and D-dimer was determined in all study patients. Data (demographics, comorbidities, type of implant, surgical procedure, microbiological results of cultures and antimicrobial treatment) of all patients were collected prospectively by the clinical researchers of the institution using a standardized data collection form.

Patients with comorbidities that could increase plasma levels of D-dimer were excluded, e.g., arterial thromboembolic disease, myocardial infarction, stroke, acute limb ischemia, intracardiac thrombus within 4 weeks, venous thromboembolic disease, such as deep vein thrombosis, pulmonary embolism, disseminated intravascular coagulation, hypercoagulation disorder, periprosthetic fracture or joint dislocation within 2 weeks, active malignancy, infections in other regions of the body, skin ulcer or hematoma.

Patients with AL were followed up after inclusion in the study for a minimum of 24 months. Clinical outcomes include infection-related failure that required a new surgery or need for antibiotic suppression. The natural progress of D-dimer in non-PJI patients was followed up either until the date of implant-related infection at any time during the first year or 1 year after revision in patients without failure.

### Statistical analysis

Statistical analysis was performed using Statistical Package for the Social Sciences Statistics for Windows version 25 (IBM Corporation, Armonk, NY, USA). Patient records were anonymized prior to analysis. Statistical analysis was performed by a physicist external to the recruitment and clinical management of the patients. Frequencies were given for categorical variables and compared in contingency tables using the Chi-squared test. Means were calculated for quantitative variables and compared using Student’s t-test. A *p*-value of less than 0.05 was considered significant. The Youden index (J = sensitivity + specificity−1) based on its correspondence with the diagnostic of PJI was used to determine the optimal threshold value for D-dimer [15]. Based on the cut-off values, the sensitivity, specificity, positive predictive value (PPV), and negative predictive value (NPV) of serum markers were calculated. The combination of different diagnostic test was performed using an “AND” combination. The combination schemes were both tests positive (test 1 positive and test 2 positive, composite diagnostic positive).

### Ethics, registration, funding, and potential conflicts of interest

The local ethics committee approved the study protocol (CTR2010.163) and all subjects provided oral informed consent before participation. This work was supported by the Fundación Marqués de Valdecilla, through the project API 11/9. IDIVAL, Instituto de Investigación Valdecilla, Cantabria. Spain. The authors have no conflicts of interest to declare.

## Results

### Study population

A total of 187 cases in 185 patients were prospectively included during the study period. 7 cases (3.8%) were excluded: 3 due to revision for dislocation, 2 due to a recent periprosthetic fracture, 1 due to a recent history of deep vein thrombosis, and 1 due to the presence of a venous leg ulcer. Of the remaining 180 cases, 141 (78.3%) were diagnosed as AL, while the other 39 (21.7%) patients were defined as PJI. Of these, 7 (18%) were classified as early, 9 (23%) as delayed, and 23 (59%) as late PJI. Detailed demographic data of all patients are presented in Table 1. There were no statistically significant differences between the 2 groups (PJI and AL) in terms of age, gender, and comorbidities (mostly diabetes) (*p* > 0.05). Patients with PJI had a greater number of previous revisions than the non-PJI group (*p* < 0.013).

**Table 1:** Characteristics of study patients

Characteristics	AL (n = 141)	PJI (n = 39)	*P *value
Age (year)-mean ± SD	69.23 ± 11.14	66.54 ± 14.13	0.220
Male n (%)	60 (42.6%)	21 (53.8%)	0.123
Body mass index-mean ± SD	30.58 ± 5.2	29.88 ± 6.41	0.542
Hip n (%)	91 (64.5%)	27 (69.2%)	0.73
Knee n (%)	50 (35.5%)	12 (30.8%)	0.73
Diabetes mellitus n (%)	25 (17.7%)	7 (17.9%)	0.86
Immunosuppressive therapy n (%) MTX	2 (2.1%)	1 (2.6%)	0.62
Systemic steroid therapy n (%)	2 (1.4%)	0	0.62
Prior revision arthroplasty n (%)	38 (27%)	20 (51.3%)	0.011
CRP mean ± SD	0.78 ± 1.57	3.47 ± 3.72	0.00011
ESR mean ± SD	15.15 ± 15.41	39.16 ± 28.40	0.000013
D-dimer mean ± SD	942.36 ± 1085.78	1968.54 ± 1471.84	0.00025

PJI, Prosthetic joint infection; AL, Aseptic loosening; MTX, Methotrexate; CRP, C-reactive protein; ESR, erythrocyte sedimentation rate; SD, Standard Deviation

Preoperative plasma D-dimer level was significantly higher in PJI cases than in AL cases (1968 ± 1471 ng/mL vs. 942 ± 1085 ng/mL; p = 0.000). Mean serum ESR and CRP values were also significantly higher among PJI patients; ESR was 39.16 ± 28.40 mm/h in the PJI group compared to 15.15 ± 15.41 mm/h in the AL group (p = 0.000), while mean CRP was 3.47 ± 3.72 mg/dL in PJI cases compared with 0.78 ± 1.57 mg/dL in AL cases (p = 0.000) (Table 1). The optimal threshold of D-dimer for the diagnosis of PJI was 1167 ng/mL, and this value demonstrated a sensitivity of 72% and specificity of 75% (75.3%), AUC = 0.774 (0.693–0.854).

### Comparison of individual diagnostic parameters for the diagnosis of PJI

According to individual parameters for the overall diagnosis of PJI, CRP achieved the highest sensitivity (84.6%), followed by ESR and D-dimer (82% and 71.8%, respectively). In early PJI diagnosis, ESR showed the highest sensibility (100%) followed by both CRP and D-dimer (85.7%). For delayed PJI, both CRP and ESR had the highest sensibility (88.8%), followed by D-dimer (66.6%) and, in late PJI, the highest sensibility was obtained with CRP (82.6%), followed by D-dimer and ESR (73.9% and 69.5%, respectively) (Table 2).

**Table 2:** Diagnostic Accuracy of individual Preoperative Tests according to type of PJI

Markers	Specificity (%)	Sensitivity (%)	PPV (%)	NPV (%)	AUC (95% CI)
D-dimer > 1167 ng/mL					
All PJI	75.3 (69–83)	71.8 (58–86)	45.9 (33.3–58.4)	90.4 (81.1–99.7)	0.774 (0.693–0.854)
Early	75.3 (69–83)	85.7 (60–100)	15.4 (4–26.7)	99 (88–100)	0.863 (0.785–0.942)
Delayed	75.3 (69–83)	66.6 (36–97)	15 (4–26.7)	97.1 (86.8–100)	0.721 (0.555–0.887)
Late	75.3 (69–83)	69.5 (51–88)	32.7 (19.5–46.8)	93.7 (83.9–1009	0.767 (0.665–0.87)
CRP > 1 mg/dL					
All PJI	82.1 (76–88)	84.6 (73–96)	56.8 (44–69)	95 (87–100)	0.869 (0.797–0.940)
Early	82.1 (76–88)	85.7 (60–100)	19.3 (5–33)	99.13 (91–100)	0.828 (0.612–1)
Delayed	82.1 (76–88)	88.8 (68–100)	24.2 (9.6–38.8)	99.1 (91–100)	0.936 (0.885–0.986)
Late	82.1 (76–88)	82.6 (67–98)	43.2 (28.5–57.8)	96.6 (88.5–100)	0.863 (0.772–0.953)
ESR > 15 mm/h					
All PJI	72.8 (65–80)	82 (70–94)	45.7 (34–57.4)	93.5 (82.8–100)	0.792 (0.7–0.885)
Early	72.8 (65–80)	100 (100–100)	15.5 (5–26)	100 (88–100)	0.897 (0.828–0.967)
Delayed	72.8 (65–80)	88.8 (63–100)	17.3 (6.4–28.3)	99 (87.3–100)	0.866 (0.765–0.968)
Late	72.8 (65–80)	73.9 (56–92)	30.9 (18.7–43.1)	94.4 (18.7–43.1)	0.767 (0.592–0.871)

PJI, Prosthetic joint infection; PPV, Positive predictive value; NPV, Negative predictive value; AUC, Area under the curve; CI, Confidence interval

### Comparison of grouped diagnostic parameters for the diagnosis of PJI

When evaluating potential combinations of 2 tests in the detection of PJI, we found that the overall combined sensitivity and specificity for ESR + CRP was 76.9% and 87.8%, respectively, 59% and 92.7% for CRP + D-dimer, and 61.5% and 88.3% for ESR + D-dimer. For each type of infection, ESR + CRP diagnosed 85.7% of early, 77.7% of delayed, and 73.9% of late cases. The combination of CRP + D-dimer diagnosed 57.1% of early, 44.4% of delayed, and 65.2% of late cases. Finally, the addition of ESR + D-dimer diagnosed 71.4% of early, 44.4% of delayed and 65.2% of late cases (Table 3). Additional file 2: Appendix S2 shows the diagnostic accuracy of individual and grouped preoperative tests in PJI.

**Table 3:** Diagnostic Accuracy of Combined Preoperative Tests according to type of PJI

Markers	Specificity (%) (95% CI)	Sensitivity (%) (95% CI)	PPV (%) (95% CI)	NPV (%) (95% CI)	AUC (95% CI)
D-dimer + CRP					
All PJI patients	92.7 (88.3–97)	59 (43.5–74.4)	69.7 (54–85.4)	88.8 (83.6–94)	0.885 (0.831–0.938)
Early	92.7 (88.3–97)	57.1 (20.5–93.8)	28.6 (4.9–52.2)	97.7 (93–100)	0.944 (0.902–0.985)
Delayed	92.7 (88.3–97)	44.4 (12–76.9)	28.6 (5–52.2)	96.2 (91.6–100)	0.914 (0.853–0.975)
Late	92.7 (88.3–97)	65.2 (45.7–84.7)	60 (40.8–79.2)	94.1 (89.6–98.5)	0.855 (0.782–0.928)
D-dimer + ESR					
All PJI patients	88.3 (82.9–93.7)	61.5 (46.3–76.8)	60 (44.8–75.2)	89 (83.5–94.4)	0.812 (0.729–0.896)
Early	88.3 (82.9–93.7)	71.4 (38–100)	23.8 (5.6–42)	98.4 (92–100)	0.917 (0.856–0.978)
Delayed	88.3 (82.9–93.7)	44.4 (12–76.9)	20 (2.4–37.5)	96 (89.9–100)	0.872 (0.786–0.957)
Late	88.3 (82.9–93.7)	65.2 (45.7–84.7)	48.4 (30.8–66)	93.8 (87.9–99.7)	0.7358 (0.633–0.882)
CRP + ESR					
All PJI patients	87.8 (82.4–93.3)	76.9 (63.7–90.1)	63.8 (50–77.6)	93.2 (87.3–99)	0.838 (0.757–0.919)
Early	87.8 (82.4–93.3)	85.7 (59.8–100)	26 (8.1–44)	99,1 (92.77–100)	0.903 (0.83–0.976)
Delayed	87.8 (82.4–93.3)	77.7 (50.6–100)	29.2 (11–47.3)	98.4 (92–100)	0.926 (0.869–0.984)
Late	87.8 (82.4–93.3)	73.9 (56–91.8)	50 (33.2–66.8)	95.3 (89.2–100)	0.783 (0.66–0.919)

PJI, Prosthetic joint infection; PPV, Positive predictive value; NPV, Negative predictive value; AUC, Area under the curve; CI, Confidence interval

### Outcome

6 (4.25%) of the 141 AL cases in our cohort had implant failure due to implant-related infection. All failures occurred during the first year after the index revision arthroplasty and, although all of them showed high levels of circulating D-dimer at the time of diagnosis, only 2 cases of early failure (< 3 months) maintained high levels of D-dimer. In both, virulent microorganisms, *Staphylococcus aureus* and *Enterobacter cloacae*, were isolated, and these patients showed raised levels of ESR and CRP after postoperative day 1 that peaked at postoperative day 4, and then remained elevated until the time of the failure, whereas plasma D-dimer showed persistently high levels that peaked at the time of the implant failure (Table 4).

**Table 4:** Aseptic loosening patients with failure due to implant-related infection

	Index revision surgery				Following revision due to infection			
Case	ESR mm/h	CRP mg/dl	D-dimer ng/mL	Time to failure (months)	ESR mm/h	CRP mg/dl	D-dimer ng/mL	Microbiology
#1	14	0.5	212	10	ND	ND	**1323**	*Staphylococcus epidermidis*
#2	5	0.2	118	1	**22**	**2.1**	**2583**	*Methicillin- sensitive S. aureus*
#3	4	0.5	768	11	7	1	**1529**	*Staphylococcus epidermidis*
#4	8	0.2	840	4	**74**	0.4	**2782**	*Staphylococcus epidermidis*
#5	10	0.4	670	2	**35**	**15.3**	**2498**	*Enterobacter cloacae*
#6	10	0.1	476	10	8	0.1	**1331**	Coagulase-negative staphylococci

Values in bold are elevated values within the reference range for each parameter

CRP, C-reactive protein; ESR, erythrocyte sedimentation rate; ND, Not done

### Microbiology and serological markers

43 microorganisms were isolated from 39 PJI patients. Only 1 patient was culture-negative and a respiratory source of infection was identified. Serological markers (ESR, CRP and D-dimer) based on the infecting organisms isolated were then evaluated (Table 5).

**Table 5:** Values of ESR, CRP and D-dimer according to microorganisms in predicting infection

Microorganisms	ESR (> 15 mm/h)	CRP (> 1 mg/dl)	D-dimer (> 1167 ng/mL)
*S. epidermidis* (n = 18)	14/18	15/18	16/18
Early	2/2	2/2	2/2
Delayed	2/3	3/3	3/3
Late	10/13	10/13	11/13
Other CoNS (n = 11)^a^	9/11	9/11	4/11
Early	2/2	2/2	2/2
Delayed	4/4	4/4	1/4
Late	3/5	3/5	1/5
*Streptococcus* spp. (n = 3)	3/3	3/3	3/3
Early	0	0	0
Delayed	1/1	1/1	1/1
Late	2/2	2/2	2/2
*Corynebacterium* spp. (n = 3)	3/3	3/3	3/3
Early	1/1	1/1	1/1
Delayed	0	0	0
Late	2/2	2/2	2/2
Gram-negative (n = 4)	3/4	3/4	3/4
Early	3/3	2/3	3/3
Delayed	0	0	0
Late	0/1	1/1	0/1
Others (n = 4)^b^	3/4	4/4	2/4
Early	1/1	1/1	1/1
Delayed	2/2	2/2	1/2
Late	0/1	1/1	0/1

ESR, Erythrocyte sedimentation rate; CRP, C-reactive protein

^a^Other CoNS (coagulase-negative Staphylococci): *Staphylococcus lugdunensis* (n = 4), *Staphylococcus capitis* (n = 3), *Staphylococcus caprae* (n = 2), *Staphylococcus haemolyticus* (n = 1), *Staphylococcus hominis* (n = 1)

^b^Others: *Enterococcus faecalis* (n = 2), *Peptostreptococcus asaccharolyticus* (n = 1)*,* methicillin-sensitive *Staphylococcus aureus* (n = 1)

## Discussion

To our knowledge, this is the first study that evaluates D-dimer levels in the diagnosis of PJI according to IDSA guidelines which highlights the use of both an abnormal ESR and CRP as the best combination for patients with suspected PJI. Our results show that plasma D-dimer did not improve the individual diagnosis of ESR or CRP for any type of PJI. The sensitivity of plasma D-dimer was lower for all PJI types, except for early infection in which it offered the second-best sensitivity, equal to ESR. When combinations of 2 tests were studied, the combined use of ESR and CRP achieved the best accuracy for all types of PJI, and the addition of D-dimer to ESR achieved the second-best sensitivity for early PJI.

In the last 2 years, D-dimer has been recommended as a promising biomarker in suspected PJI. Indeed, D-dimer was introduced in 2018 as an alternative biomarker with a cut-off value of 860 ng/mL in the new MSIS and ICM validated diagnostic PJI criteria [5, 6]. Recently, 8 meta-analyses addressing the diagnostic accuracy of D-dimer in PJI highlighted the wide heterogeneity among studies, because of that, univariate meta-regression and subgroup analysis were performed and found that the type of sample (serum or plasma) could be the primary factor affecting the heterogeneity of sensitivity, while the country of origin of the study (China and USA) may be the main source of heterogeneity in terms of specificity [16–23].

Seven studies have investigated plasma D-dimer in the diagnosis of PJI, including 6 Asian studies and 1 European study (Germany) (Table 6) [24–30]. On the basis of our study conducted according to IDSA guidelines, plasma D-dimer achieved the second-best sensitivity and the third-best specificity when compared with the aforementioned studies. Our results are in close agreement with those published in Asian populations, in terms of both sensitivity and specificity, and contrast with the European study. In this regard, 2 studies on serum D-dimer that recruited their patients from an American population using MSIS criteria and a threshold value of 850 ng/mL showed conflicting results, with sensitivities and specificities of 89% and 93% vs 96% and 32%, respectively [7–10]. Therefore, it should be reevaluated whether racial differences are a major factor affecting the outcome of the different studies.

**Table 6:** Plasma D-dimer Studies

References	Country	PJI/AL	Study design	Site	Age (PJI/AL)	BMI (PJI/AL)	Cut-off ng/mL	Standard Reference	Sensitivity (%)	Specificity (%)	Exluded hipercoagulation disorder	Excluded inflammatory arthritis
Fu et al. [24]	China	15/15	Prospective	H/K	65.6/65.4	25.7/24.7	850	MSIS	66.67	60	No	Yes
Li et al. [25]	China	76/363	Retrospective (multicentre)	H/K	63.7/61.3	25.0/25.1	1250	ICM	64.5	65	No	No
Xu et al. [26]	China	129/189	Retrospective	H/K	NA	NA	1020	MSIS	68.29	50.70	No	No
Chen et al. [30]	China	30/60	Retrospective (Matched study)	H/K	61.5/67.7	26.0/24.9	1690	MSIS	66.7	76.7	No	No
Wu et al. [27]	China	35^a^27^b^/81	Retrospective	H/K	62.6/69.1	24.7/24.5	410	MSIS	75.8	67	No	No
Ackmann et al. [28]	Europe (Germany)	52/67	Retrospective	H/K	70.5/68	30.9/28.4	2750	MSIS	38	94	Yes	Yes
Huang et al. [29]	China	47/38	Retrospective	H/K	63.7/66.8	NA	1170	MSIS	60	85	Yes	Yes
Present study	Europe (Spain)	39/141	Prospective	H/K	69.2/66.5	30.6/29.9	1167	IDSA	71.8	75.3	Yes	No

PJI, Prosthetic joint infection; AL, Aseptic loosening; MSIS, Musculoskeletal Infection Society; ICM, International Consensus Meeting; IDSA, Infectious Diseases Society of America; NA, not applicable; H, Hip; K, Knee

^a^Patients underwent first-stage and spacer insertion. ^b^Patients underwent second-stage reimplantation

Among other concerns, the optimal cut-off value for D-dimer and its sensitivity and specificity remain unclear. Some authors considered that patients’ body mass index (BMI), age or sex may comprise a confounding factor resulting in higher levels of D-dimer, while women and older patients may have higher serum levels of D-dimer when used in the diagnosis of thrombosis and/or embolism [31]. In our cohort, the threshold for plasma D-dimer was similar to that described in the Asian population, particularly by Huang et al. [29], who also reported lower mean BMIs and lower median age than the Europeans. Recently, Grzelecki et al., found that the influence of the operated joint type may be a very important factor in the concentration of D-dimer and that plasma D-dimer has a relatively high value in the detection of knee PJI, but a moderate to low value for hip PJI [32]. It does seem logical that these characteristics could have unbalanced the optimal threshold of D-dimer for the diagnosis of PJI which ranged between 410 ng/mL to 2300 ng/mL. In short, pending more robust studies that provide new data and investigate their correlations with sample type, comorbidities, ethnic variability, joint type, PJ type, etc., each institution should optimize D-dimer thresholds.

A further point to be discussed is whether plasma or serum is the most appropriate sample for performing D-dimer testing. The coagulant factor present in plasma but not in serum interferes with many biochemical parameters and the higher metabolite concentrations in serum offer more sensitive results in biomarker detection. Korte et al. demonstrated a close agreement of D-dimer concentrations determined from citrated plasma samples and serum for the exclusion of venous thromboembolism [33]. However, they added that the use of serum for D-dimer determination needs to be evaluated and validated in a clinical study. A recent meta-analysis of prosthesis-related infections showed that serum D-dimer outperformed plasma D-dimer in sensitivity and specificity (0.86 and 0.84 vs. 0.67 and 0.60, respectively) [34]. In this regard, 6 studies on serum D-dimer in PJI were published between 2017 and 2020, 2 from the USA and 4 from China. Five used MSIS criteria for the diagnosis of PJI and the other used ICM. The D-dimer threshold ranged from 850 to 1170 ng/mL [7–12]. Unfortunately, they reached different conclusions and these results raise the question of whether differences in the type of sample are a major factor affecting the substantial discrepancies between studies.

In a prospective study that measured D-dimer levels before and after primary total hip or knee arthroplasty, the most significant changes in D-dimer levels were observed during the early postoperative period. Levels increased sharply and peaked on the first day after joint replacement surgery, decreasing to baseline levels on the following day [35]. In our AL cohort, 4.25% of the cases had implant failure due to infection. All of them had high levels of circulating D-dimer at the time of diagnosis. However, in the 2 cases with early failure, D-dimer levels remained raised and peaked at the time of failure. In these 2 cases, ESR and CRP levels peaked before the time failure was diagnosed. It would be of interest to study a larger number of cases with early failure to determine if there is any difference.

Finally, coagulase-negative staphylococci (CoNS) (67.4%) were the most common pathogens isolated in our study, while *S. epidermidis* was the most prevalent species, particularly in late infections. Higher plasma D-dimer levels were associated with a slight tendency towards the detection of more *S. epidermidis* isolates than other inflammatory markers, but in contrast were associated with fewer PJI infections due to other CoNS isolates. We are unaware if small colony variants or biofilm formation by different CoNS play a role in these findings. No differences were found for other isolates such as low-virulence bacteria.

Our study has several limitations that should be considered. First, this is a single-center prospective study with potential for uncontrolled selection biases; however, methodologies were applied in a standard fashion, something that does not occur when patients are included from multiple centers where the test result of D-dimer can be interpretated as positive or negative with different thresholds and, therefore any change in thresholds may have a substantial impact on its diagnostic value. Second, the sample size for PJI infections in the study was small due to the low incidence of PJI and may lack statistical power to detect some associations in different subtypes of PJI being this limitation is described in other studies [30]. Third, there is no gold standard for the diagnosis of PJI and it is possible that the lack the sensitivity in detecting PJI, specially in chronic or low-grade infections. Fourth, we excluded patients with conditions that may induce high expression of D-dimer and could result in high false positive rates. These pathologies accounted for almost 4% of the study patients and, this low prevalence, does not appear to have been a bias for the study. However, different D-dimer assays may point to differences in sensitivity and specificity between studies. This emphasizes the need for standardization of D-dimer assays. Fifth, prior revisions of PJI group differed significantly from AL group which may affect the validity of the results. It has been recognized that the risk of PJI increases with the number of previous joint arthroplasties. However, it has been unclear whether the increased risk of PJI in patients with prior joint arthroplasties is due to an increased number of comorbid conditions, a prolonged operating time, an increased number of blood transfusions, or higher frequency of postoperative wound complications. Therefore, the identification of patients at high risk for PJI would allow for improved preoperative risk assessment, increase the index suspicion, and identify patients for whom focused efforts at prevention are necessary.

In conclusion, plasma D-dimer determined according to IDSA guidelines did not improve the individual or combined diagnosis of ESR or CRP for any type of PJI. The persistence of raised levels of plasma D-dimer after revision arthroplasty in AL cases might be used effectively in diagnosing early postoperative infection. Because of the remaining concerns surrounding this new serological biomarker in implant-associated infection, we urge caution in accepting serum and/or plasma D-dimer as a first-line screening test for PJI diagnosis, regardless of the guidelines adopted. This conclusion should be confirmed in appropriate clinical trials.

## Supplementary Information

**Additional file 1: Appendix S1.** Histopathological classification of the periprosthetic membrane.

**Additional file 2: Appendix S2.** Diagnostic Accuracy of individual preoperative tests and combinated test in PJI. *PPV* Positive predictive value; *NPV* Negative predictive value; *AUC* Area under the curve; *CI* Confidence interval.

## Data Availability

The database used and/or analysed during the current study are available from the corresponding author on reasonable request.

## Abbreviations

AL: Aseptic loosening
AUC: Area under the Curve
BMI: Body mass index
CoNS: Coagulase-negative staphylococci
CRP: C-reactive protein
ESR: Erythrocyte sedimentation rate
ICM: International Consensus Meeting
IDSA: Infectious Diseases Society of America
MSIS: Musculoskeletal Infection Society
NPV: Negative predictive value
PJI: Prosthetic Joint Infection
PPV: Positive predictive value
ROC: Receiver operating characteristic
