# Supplementary material for: The poor accuracy of D-dimer for the diagnosis of prosthetic joint infection but its potential usefulness in early postoperative infections following revision arthroplasty for aseptic loosening
Source: BMC Infect Dis. 2022 Jan 27;22:91. doi: 10.1186/s12879-022-07060-8 (PMC8793264; doi:10.1186/s12879-022-07060-8)
Supplement: Supplementary file 1 — Additional file 1: Appendix S1. Histopathological classification of the periprosthetic membrane. [file 12879_2022_7060_MOESM1_ESM.docx]

| Type I. Wear particle induced type | Detection of foreign body particles; macrophages and multinucleated giant cells occupy at least 20% of the area |
| --- | --- |
| Type II. Infectious type | Granulation tissue with neutrophilic granulocytes, plasma cells and few, if any, wear particles. |
| Type III. Combined type | Aspects of type I and type II occur simultaneously |
| Type IV. Indeterminate type | Neither criteria for type I nor type II are fulfilled |

Appendix 1. Histopathological classification of the periprosthetic membrane.

Morawietz L, et al. Proposal for a histopathological consensus classification of the periprosthetic interface membrane. J Clin Pathol. 2006;59(6):591–7.
