# Supplementary material for: The poor accuracy of D-dimer for the diagnosis of prosthetic joint infection but its potential usefulness in early postoperative infections following revision arthroplasty for aseptic loosening
Source: BMC Infect Dis. 2022 Jan 27;22:91. doi: 10.1186/s12879-022-07060-8 (PMC8793264; doi:10.1186/s12879-022-07060-8)
Supplement: Supplementary file 2 [file 12879_2022_7060_MOESM2_ESM.odt]

Appendix 2. Diagnostic Accuracy of individual preoperative tests and combinated test in PJI

|  | D-dimer> 1167ng/ml | CRP >1 mg/dL | ESR >15mm/h | CRP+ESR | D-dimer+ CRP | D-dimer+ ESR |
| --- | --- | --- | --- | --- | --- | --- |
| Specificity | 75.3 (69%-83%) | 82.1% (76%-88%) | 72.8% (65%-80%) | 87.8% (82.4%-93.3%) | 92.7% (88.3%-97%) | 88.3% (82.9%-93.7%) |
| Sensitivity | 71.8% (58%-86%) | 84.6% (73%-96%) | 82% (70%-94%) | 76.9% (63.7%-90.1%) | 59% (43.5%-74.4%) | 61.5% (46.3%-76.8%) |
| PPV | 45.9% (33.3%-58.4%) | 56.8% (44%- 69%) | 45.7% (34%-57.4%) | 63.8%% (50%-77.6%) | 69.7% (54%-85.4%) | 60% (44.8%-75.2%) |
| NPV | 90.4% (81.1%-99.7%) | 95% (87%-100%) | 93.5 (82.8%-100%) | 93.2% (87.3%-99%) | 88.8% (83.6%-94%) | 89% (83.5%-94.4%) |
| AUC (95%CI) | 0.774 (0.693-0.854) | 0.869 (0.797-0.940) | 0.792 (0.7-0.885) | 0.838 (0.757-0.919) | 0.885 (0.831-0.938) | 0.812 (0.729-0.896) |
